# Red Beet Extract Powder, Gelatin and Sucrose Interactions in Gummy Candies

**DOI:** 10.3390/foods14173138

**Published:** 2025-09-08

**Authors:** Omer Said Toker, Ilyas Atalar, Abdullah Kurt, Ibrahim Palabiyik, Nevzat Konar

**Affiliations:** 1 Food Engineering Department, Faculty of Chemical and Metallurgical Engineering, Yıldız Technical University, İstanbul 34220, Türkiye; stoker@yildiz.edu.tr; 2 Food Engineering Department, Agriculture Faculty, Eskisehir Osmangazi University, Eskisehir 26040, Türkiye; ilyas.atalar@ogu.edu.tr; 3 Food Engineering Department, Aksehir Faculty of Engineering and Architecture, Selcuk University, Konya 42130, Türkiye; 4 Food Engineering Department, Agriculture Faculty, Tekirdag Namik Kemal University, Tekirdag 59030, Türkiye; ipalabiyik@nku.edu.tr; 5 Dairy Technology Department, Agriculture Faculty, Ankara University, Ankara 06100, Türkiye; konar@ankara.edu.tr

**Keywords:** confectionery, gelatin, sucrose, colorant, optimization

## Abstract

Gummy candies rely on sugar, gelatin, and synthetic colorants, but rising demand for natural alternatives makes replacement essential. Interactions between natural additives and main components (gelatin, sucrose), especially their effects on color and texture, remain unclear. This study examines the existence and amounts of sucrose, gelatin, and natural coloring agent, red beetroot extract powder (RBEP), in gummy candy compositions, along with main quality attributes and stability behavior. The effects of these variables on the main physicochemical, color, texture and bioactive properties were investigated. Model gummy samples’ hardness (10.68–19.18 N), resilience (0.57–0.89), cohesiveness (0.87–1.01), springiness (0.19–0.54 mm), gumminess (9.63–21.30 N), and chewiness (2.15–8.29 N × mm) properties were determined by texture profile analysis. The values of L*, a*, b*, chroma, and hue angle were determined in the ranges of 23.9–91.5, (−0.93)–43.6, 1.06–8.17, 6.20–44.0, and 5.97–97.4, respectively. The interactions between RBEP × gelatin and RBEP × sucrose were found to be effective in color parameters. Total phenolic content (TPC) values and inhibition values for antioxidant activity (AA, % inhibition) ranged from 25.6 to 257.4 mg GAE/kg and 0.00–49.8%, respectively. The optimum composition, determined by considering texture properties and stability behavior as the response, revealed the concentrations of sucrose, gelatin solution, and RBEP as 34.53 g/100 g, 18.33 g/100 g, and 0.44 g/100 g, respectively. The study is the first to investigate the effect of RBEP concentration on the quality parameters of gummy candies and interactions with other components of the formulation. The results will raise awareness about the use of colorants in the confectionery industry and contribute to developing novel products.

## 1. Introduction

The widespread use of synthetic additives in confectionery products has raised increasing health concerns worldwide [1], as artificial colorants commonly found in candies marketed to children have been linked through toxicological and clinical studies to behavioral changes and hypersensitivity reactions [2,3]. Natural food colorants, therefore, have attracted attention not only for their visual and sensory appeal but also for their potential health-promoting properties. Natural coloring agents, frequently used in gummy candies and compatible with the flavors commonly found in their composition, enhance the perception intensity of these flavors through visual characteristics. However, due to the changing consumer trends and expectations, reducing ingredients such as sugar, gelatin, artificial flavors, and colorants in candies gained importance [4].Therefore, it is essential to explore the possibilities of using natural colorants in gelatin-based candy compositions, characterize their effects on major quality attributes, and understand their interactions with other components.

One of the commonly used natural colorants is obtained from red beetroot. Red beetroot (*Beta vulgaris* L.) belongs to the Amaranthaceae family and can be cultivated under various climatic conditions. Beetroot is grown in temperate climates throughout Asia, America, and Europe [5]. The extract obtained from the plant, or the powder obtained especially through the spray-drying method, is commonly used as a natural colorant in various foods [6]. Beetroot peel is a valuable source of dietary fibers and betalain pigments with strong antioxidant activity, linked to the prevention of cardiovascular and neurodegenerative disorders. The characteristic red color of beetroot mainly derives from its high betalain content (≈200 mg/100 g fresh weight) [7,8]. Its major application in the food industry is as a juice concentrate used for coloring and as a source of the natural additive betanin dye (E162) [5]. Therefore, the valorization of beet by-products offers a promising strategy to reduce food waste, alleviate food insecurity, and mitigate environmental pollution. It is used in various foods, including bakery products subjected to high temperatures, low-temperature treated foods such as ice cream, and widely consumed foods like yogurt and candies [9,10,11,12,13]. Hence, it is important to consider the interactions between red beetroot extract powder (RBEP) and various food matrices, as well as the major and minor components within these matrices. For example, the color and hues of various colorants and pigments are affected by product pH and matrix. Hence, chosen colorants need to be stable at the pH levels of the products and display the desired color characteristics. Additionally, interactions between product components and colorants should be considered. For instance, gelatin-based candies are generally characterized by low pH and water activity, suitable for sufficient anthocyanin stability [14]. However, using ascorbic acid in this composition may lead to the destabilization of anthocyanins [15], or interactions between pigments and flavor compounds may cause deviations in product quality properties [16]. Therefore, determining the relationships between these interactions and quality and stability behaviors would be beneficial.

In gummy candies, the major components include sucrose, corn syrups, and gelatin [17]. Due to its high molecular weight, gelatin can form a three-dimensional network after forming triple helix structures and junction zones. The quantity of junction zones escalates as gelation advances. Temperature, pH, ash content, concentration, and interactions with other food components affect gelatin gelation or its glass transition temperature (Tg) [18] Therefore, the interaction between colorants and gelatin and the consequences of this interaction should also be considered. For instance, Schrieber and Gareis [19] highlighted the interaction between gelatin and polyphenols as a cross-linking that affects the water solubility of gelatin, suggesting theoretically higher bloom gelatin usage in conditions where polyphenols are present. Furthermore, when used as a hydrocolloid, the gelatin network’s structure determines the candies’ physical properties [20]. Both the processing conditions and the candy composition are of great importance for the textural properties of soft candies due to their influence on the network formation rate and the molecular network’s subsequent strength. The gelatin type and concentration significantly affect the properties of gummy candies. Sucrose is another essential component that influences the characteristic features of candies. For example, different candy products emerge depending on the presence and quantity of sucrose crystals [21]. It has been determined that the interaction between gelatin and sucrose affects the final product texture properties, and it should be considered for forming the gelatin network [17]. Therefore, it may be necessary to determine the indirect interactions of components that may affect the gelation behavior of gelatin with sucrose as well.

This study aims to determine the effects of RBEP in gummy candies, considering interactions with other major components, and characterizing the stability properties of these interactions. For this aim, gummy candy samples containing different amounts of sucrose, gelatin solution, and RBEP were prepared using the mixture design method. Texture properties (hardness, resilience, springiness, cohesiveness, gumminess, and chewiness) were determined in the prepared samples, and the effects of independent variables, such as sucrose, gelatin solution, and RBEP amounts, as well as their interactions, on the main physicochemical properties (dry matter content, pH, water activity), color (L*, a*, b*, C*, hue angle), were investigated. The aim was to determine and interpret the effects of these interactions. The main texture parameters that stand out in soft confectionery products used for categorization include hardness and springiness. These properties are related to the functional activities of gelatin [4]. Additionally, certain colorants, including RBEP, can affect gelatin’s functions in the confectionery structure [22]. Due to these interactions, maximum hardness and springiness, minimum ΔE* value and hardness and springiness changes were targeted for formulation optimization. In addition to the samples’ total phenolic content and antioxidant activity, changes in color and texture properties under storage conditions were also investigated.

## 2. Materials and Methods

### 2.1. Materials

Water, sucrose (Konya Sugar, Konya, Turkey), 40–42 DE (dextrose equivalent) glucose syrup (Sunar, Adana, Turkey), gelatin (220–250 bloom) (Gerede Jelatin, Bolu, Turkey), RBEP (Sensient, Kocaeli, Turkey) were used in the preparation of candy samples.

### 2.2. Study Design

The D-optimal mixture design method was used in the optimization process. Gelatin solution (220–250 Bloom, Type A, Bovine Gelatin) (15.0–20.78 g/100 g), sucrose (32.0–37.76 g/100 g), and RBEP (0.00–1.00 g/100 g) amounts according to preliminary test results as independent variables.

### 2.3. Sample Preparation

Gummy candy samples were prepared with water, sucrose, 42 DE glucose syrup, gelatin solution, and natural coloring agent (RBEP) (Table 1). Samples with determined optimum gummy composition and a control sample were produced for sensory evaluation. Previously used compositions without any coloring agent were considered for control sample composition. Water, sucrose, and glucose syrup were mixed and heated to 100 °C for 10 min in a thermal mixer (Thermomix TM5, Vorwerk, Dessau-Roßlau, Germany) to achieve 85°Bx [23]. Subsequently, the mixture was cooled to 90 °C. Previously prepared gelatine: water solution (1:3) was included in the mixture and stirred at 90 °C for 2 min. After adding RBEP, the mixture was quickly cooled to 60 °C and mixed again (1 min, 200–300 rpm). The syrup was transferred to silicone molds and rested at 20 °C for 24 h. 500 g batches were prepared for each experiment.

### 2.4. Physico-Chemical Analysis

The dry matter content (DMC) was determined gravimetrically in an incubator at 60 °C until constant weight [24]. Water activity values were measured by a water activity analyzer (Aqualab 4TE, München, Germany) at 20.0 °C. pH values were measured by a pH meter (Ohaus, AquaSearcher, Fairfield, NJ, USA). The total soluble solid content was measured with a hand-type refractometer (Abba, Atago, Tokyo, Japan).

### 2.5. Texture Analysis

Texture Profile Analysis (TPA) of the candy samples was determined using a TX-XT plus device (Stable Micro Systems, Godalming, UK). Candy samples removed from the molds had dimensions of 28 mm in diameter and 20 mm in height. A cylindrical probe with a diameter of 35 mm and a 5 kg load cell were included with the device. There was a 15-s gap between the two consecutive 50% compressions. The test was conducted at a speed of 1 mm/s. Hardness, springiness, cohesiveness, gumminess, chewiness, and resilience values were computed using the test’s force-time curve. Five replicates of each sample were conducted.

### 2.6. Antioxidant Capacity and Total Phenolic Content

#### 2.6.1. The 2,2-Diphenyl-1-picrylhydrazyl (DPPH) Method

The method of Brand-Williams et al. [25] was used to calculate the DPPH antioxidant capacity. One gram of the sample was extracted with 10 mL of 80% methanol solution for 5 min. The mixture was centrifuged at 3000× *g* for 15 min at 25 °C. 100 μL of the supernatant was mixed with 3900 μL of the DPPH solution and kept for 1 h in dark conditions. The absorbance at 517 nm DPPH activity was calculated using Equation (1).

(1) $$Antiradical\;activity\left(\%\right)=\left[\left(A_{C}-A_{T}\right)/A_{C}\right]\times 100$$

where *Ac* = absorbance value of control and *A_T_* = absorbance value of sample

#### 2.6.2. Total Phenolic Content

Gummy candy’s total phenolic content (TPC) was calculated according to Cao et al. [26]. A mixture of 3.16 mL distilled water, 200 μL Folin-Ciocalteu reagent solution, and 600 μL of 20% sodium carbonate was added to the sample extract and kept at room temperature for 2 h. The mixture’s absorbance value at 765 nm was measured with a spectrophotometer (Jasco V-730, Tokyo, Japan).

### 2.7. Color Analysis

The CIE-Lab method was used to analyze the color characteristics of gummy samples using a colorimeter (CR-4000, Konica Minolta, Tokyo, Japan). After measuring the samples’ L* (luminance), a* (±red-green), and b* (±yellow-blue) values, the chroma (C*) and hue angle (h*) values were calculated. The chroma (C*), hue angle (h*), and color stability (ΔE) values were calculated by using Equations (2)–(4). Five replicates were performed for each treatment.

(2) $$C^{*}=\sqrt{{a^{*}}^{2}+{b^{*}}^{2}}$$

(3) $$h^{*}={tan}^{-1}\left(\frac{b^{*}}{a^{*}}\right)$$

ΔE* = [(ΔL*)^2^ + (Δa*)^2^ + (Δb*)^2^]^1/2^ (4)

### 2.8. Storage Stability

Using the procedure outlined by Subramaniam [27], gummies without packaging were put in a shelf-life cabinet and stored at testing conditions (25 °C and 70% relative humidity). Color (Section 2.6) and texture profile analysis (Section 2.4) were done at 7-day intervals. The initial measurements were used to calculate the values of ΔL*, Δa*, and Δb*. Furthermore, a percentage change (%) was calculated between the TPA measurements at the beginning and the end of 90 days of storage.

### 2.9. Sensory Evaluation

A total of 40 volunteer panelists (graduate, bachelor, and master’s students; aged 20–30 years; non-smokers and without known food allergies) from the Food Engineering Department of Eskişehir Osmangazi University participated in the sensory evaluation. The test was conducted in a sensory analysis laboratory under standardized conditions (individual booths, white lighting, and 22 ± 2 °C). Control and optimum gummy samples were coded with random three-digit numbers and presented to panelists in a randomized order. Water was provided for palate cleansing between samples. Each attribute, including appearance, taste, elasticity, softness, and overall acceptability, was evaluated using a 9-point hedonic scale, where 9 indicated “like extremely” and 1 indicated “dislike extremely”. Evaluations were performed in two replications to ensure reproducibility.

### 2.10. Statistical Analysis

The mean ± standard deviation was calculated for the results of the analyses conducted on all samples. All analyses were done in triplicate, and the significant terms in the model were determined by analysis of variance (ANOVA). The lack-of-fit test, and determination of the regression coefficients were applied to determine the adequacy of the optimization models (Supplementary Tables S1–S4). Design Expert (Stat-Ease Inc., trial version 13.0, Minneapolis, MN, USA) was used for statistical analysis and modeling. Variances were considered statistically significant at *p* < 0.05. Confirmation tests were carried out to check the precision and accuracy of the results. The findings were found between 95% prediction intervals after comparing the experimental and predicted values to assess the models’ validity. The independent samples *t*-test was used to evaluate the sensory analysis results. Pearson’s correlation analysis test was applied to determine the correlation between the responses by using the SPSS program for Windows (version 21.0; SPSS Inc., Chicago, IL, USA).

## 3. Results and Discussion

### 3.1. Physico-Chemical Properties

The total soluble solid content (TSS) is a critical physicochemical characteristic in gummy candies. The TSS values of gummy samples syrups prepared with RBEP ranged from 84.1 to 89.2°Bx (Table 1). The concentrations of sucrose and gelatin solution, independent variables in the study, are expected to influence the TSS values. For instance, samples using a high amount of gelatin solution initially contained more water. However, as shown in Supplementary Table S1, an important model about the impact of these variables on this characteristic couldn’t be identified (*p* < 0.05). However, slight negative interactions were identified between TSS and texture properties, with mild impacts on hardness and chewiness and weak effects on springiness and chewiness (Table 2). This situation might again be associated with the variation in the amount of gelatin solution used. Molina-Rubio et al. [28] reported that the variety of hydrocolloids in the syrup formulation and its Brix value affected the hardness property of sugar syrups. However, no effect of coloring concentration was determined. In gummy candies, by altering processing conditions, mainly focusing on optimizing the impact and quantity of hydrocolloid concentration, deviations in quality properties arising from innovative components or reformulation studies are often tolerated [29]. 

Water content plays a pivotal role in the manufacturing, texture, and storage stability of confectionery products. In sugar-based confections, reduced moisture levels generally lead to higher dry matter contents, which contribute to firmer textures and extended stability [30]. Confectionery products subjected to heat treatment at high temperatures have lower water content, hence higher dry matter content. If the moisture content is not suitable for the type of sugar used, the confectionery obtained can be either too soft or too hard. Additionally, high moisture content results in soft confectionery. Therefore, the samples’ DMC is critical. For gummy samples prepared using different sucrose, gelatin solution, and RBEP levels, the dry matter contents range from 78.8 to 82.8 g/100 g (Table 1). These values were higher than 76.0 g/100 g, which could be a critical limit for the relevant product group. In the context of gummy candies, Cebin et al. [31] reported dry matter contents and water activity levels that closely match ours, reinforcing the critical role of dry matter in textural properties and product stability. Additionally, Tireki et al. [32] demonstrated that formulation variables and storage temperature notably affected the moisture content, pH and textural behavior of gummies. Dry matter content (DMC) values above 76 g/100 g should be achieved to inhibit microbial growth in confectionery products [33]. To prevent crystallization at these levels, a certain amount of sucrose is substituted with other sugars (e.g., glucose syrup or invert sugar) to increase solubility. 

The DMC varies depending on different factors and agents, weakening the texture of products containing gelatin. Therefore, adjusting the DMC using bulking agents alongside sugar substitution is necessary. Molina-Rubio et al. [28] determined that hydrocolloid type in sugar syrups and their Brix value influenced the hardness property. The impact of independent variables on DMC has also been modeled as quadratic (*p* = 0.0024) with an R^2^ value of 0.8072 (Supplementary Table S1). However, interactions between variables were insignificant (*p* < 0.05). It must also be considered one of our study’s shortcomings. To manufacture gummy samples, gelatin must be used as a gelatin solution (Gelatin: water, 1:3). Furthermore, following heat treatment, gelatin solution should be included in the main mixture [33]. Consequently, in research where the gelatin solution is a variable, the initial total water content may vary with each trial. Gummy formulations’ water content (5.1 g/100 g) was maintained throughout our study. However, depending on the used gelatin solution (15.00–20.78 g/100 g), there was a minor difference in the amount of water necessary to prepare the samples (15.46–19.47 g/100 g). To minimize this potential effect, the syrup was heated to a specific TSS value (85°Bx) before the gelatin solution was added [23].

The research found moderate to strong connections between the samples’ hardness, springiness, cohesiveness, chewiness, and water activity properties and their DMC. This correlation’s coefficient values (r) ranged from −0.471 to −0.712 (Table 2). In particular, the correlations between texture properties and DMC are critical. Changes in moisture content strongly influence confectionery quality by altering texture (hardness, stickiness), storage stability, and sensory acceptability, as previously reported for sugar- and gelatin-based products. In particular, inadequate moisture control can result in either overly soft or excessively hard products, directly affecting consumer perception and product storage stability [34,35]. High moisture content can induce internal molecular mobility in confectioneries [30]. Additionally, water can act as a plasticizer. The breakdown of hydrogen bonds between water molecules and the cross-linking of gelatin chains may create new hydrogen bonds between gelatin and water molecules when the product’s moisture level decreases [35]. The release and perception of aroma components are also influenced by moisture content, particularly in gummy candy. Polar compounds are held at lower levels and diffuse quickly as they are more soluble in water. Low molecular weight sugars can alter the amount of free water with solvent characteristics, impacting these polar molecules’ release and diffusion [36]. Therefore, it is crucial to determine the impact of RBEP, gelatin solution and sucrose on dry matter content, especially in gummy candies.

Water activity is important for assessing the available water content in food composition and its microbial stability and shelf life [37]. In our study, the effects of independent variables on water activity were investigated, and values for the property ranging from 0.66 to 0.74 were determined for the samples (Table 1). These results are generally consistent with the values expected for gummy candies [33]. Additionally, an important model was identified for the changes in water activity concerning the effects of independent variables (*p* < 0.05). However, the model’s R^2^ value was relatively low (0.3696) and *p* value (0.0498) was relatively high and close to 0.05. For this reason, the model could be defined as non-decisive.

An increase in the amount of sucrose led to a decrease in water activity, while higher levels of gelatin solution resulted in samples with higher water activity (Supplementary Figure S1). No significant effect of RBEP concentration on water activity was determined. Previous studies have emphasized that increased sucrose concentration can reduce water activity values. In our study, it was determined that there is a correlation between water activity values and hardness (0.562), resilience (0.486) and chewiness (0.582) properties, as expected. However, the correlation (−0.451) findings show that an increase in the a* value may reduce water activity, and the determination of a similar correlation for chroma values (−0.467) was also remarkable (Table 2). Although a relationship with the amount of coloring used was not determined, a moderate negative correlation was found between the water activity and the color properties directly resulting from these colorants.

Gummy candies are primarily consumed for sensory satisfaction [38]. Therefore, flavor characteristics and the factors influencing these characteristics are important in gummy candy technology. For example, it has been determined that citric acid, which affects flavor perception, and thus pH, also affects the texture properties of gelatin gels [39]. Therefore, changes in processing conditions may provide advantages in altering texture properties. pH values not only affect color stability but also influence the behavior of colorants in the matrix. This study determined the pH values of gummy candy samples between 4.71 and 5.74 (Table 1). The lack of citric acid throughout the sample preparation resulted in comparatively high pH values. Gummies have a pH between 3.00 and 5.00, which includes food acids, particularly citric acid [40]. For example, pH of gummies was reported to be 3.15–5.00 by Ge et al. [41]. However, Wang and Hartel [39] found that for samples that did not contain a food acid, this value was between 5.00 and 5.30. A significant linear model was determined for the effect of independent variables on pH values (*p* < 0.05), with an R^2^ value of 0.8267 for this model (Supplementary Table S2). While sucrose concentration did not affect pH, the effect of gelatin solution amount on pH increase was considered negligible. However, it was found that an increase in RBEP amount resulted in a significant decrease in pH (Supplementary Figure S2). In parallel with these results, a strong and negative correlation (−0.889) existed between pH value and RBEP concentration (Table 2).

### 3.2. Texture Characteristics

Candies are among the foods where textural properties significantly impact oral experiences and perception [42]. Therefore, textural properties are among the primary quality indicators and are decisive in consumer acceptance and preference. The texture in candies results from the interactions of various components and structural elements at macro- and micro-levels [43]. Considering their sensorial importance, textural parameters in confectionery studies typically include hardness, cohesiveness, springiness, resilience, chewiness and gumminess [7]. In this study, the hardness (10.63–19.18 N), resilience (0.57–0.89), cohesiveness (0.87–1.02), springiness (0.19mm–0.54mm), gumminess (9.62–19.14 N), and chewiness (2.15–8.29 N × mm) values of gummy samples were determined through texture profile analysis (Table 3). Significant models were identified for TPA (Texture Profile Analysis) parameters except for springiness and chewiness (Supplementary Table S3), which are generally linear models (*p* < 0.05). It was observed that the concentrations of sucrose and gelatin significantly influenced the TPA values at moderate to high intensities, while the amount of RBEP used had tolerable effects (Supplementary Figure S1).

Confectionery products rely critically on sugar crystallization for suitable texture, appearance, and storage stability. While sucrose crystals are desired in some confectionery products (such as fondant, toffee, and fudge), they are undesirable in gummy candies [21]. For instance, a significant function of glucose syrup, commonly used in gummy candies, is to inhibit sucrose crystallization. In chewy candies like gummies, high hardness can adversely affect product chewiness and texture, which is an undesirable trait [44]. However, candies’ low hardness and easy penetration indicate a weak gel structure [45]. This study determined that an increase in the amount of sucrose led to a decrease in the hardness of gummy samples. Increasing the concentration of the gelling agent, decreasing the pH, utilizing glucose syrup with a low DE (dextrose equivalent) value, or decreasing the sucrose/glucose syrup ratio are some methods to increase instrumental hardness [17].

Cohesiveness, which does not correlate with other TPA parameters, indicates the strength/rigidity of internal bonds [37]. For instance, Dalabasmaz et al. [23] observed interactions between mixing rate after gelatin and TPA parameters, except for cohesiveness, for model bovine gelatin gels, including sucrose and glucose syrup. Additionally, Zhang et al. [46] reported that while cohesiveness and springiness remained relatively the same, hardness increased for gels and food samples, including these gels. This study determined that an increase in sucrose content led to a decrease in cohesiveness values, while an increase in gelatin content resulted in high cohesiveness (Supplementary Figure S1). However, it was determined that the concentration of coloring had no significant effect on this TPA parameter. Therefore, the effect of coloring concentration on gelatin’s cohesive impact could not be established.

Reduction in cross-links between gelatin molecules may decrease the cohesiveness of gummy candy [35]. In this regard, it was observed that RBEP did not affect gelatin cross-linking. Different components showed varying degrees and directions of effects. For example, Molina-Rubio et al. [28] reported that the brix value also influences the cohesive work in semi-solid matrices obtained by gelatinization of sugar. Also, a weak correlation (0.30 < r < 0.50) was identified in this study between DMC and cohesiveness. This result should be evaluated considering the confectionery’s stickiness property increasing with moisture content and the effect of corn syrup ratio to reach the highest stickiness level [47].

Chewiness is a texture parameter examined in semi-solid foods. Periche et al. [43] found that the most significant influence on textural parameters like chewiness and hardness comes from variations in the gelatin used in product formulation. They also noted that sugars and their combinations impact these mechanical properties. Adding sugars and the presence of these substances can lead to changes in the structure formed by solid materials [40]. Parallel results concerning this relationship were obtained in the study. Gelatin’s main technological functions include gelling, thickening, stabilization, texture regulation, and emulsification [48]. However, the presence and concentrations of sugars in the composition affect gelatin functions because sugars affect the stabilization of gelatin gel configuration [17]. Sucrose supports gelatin solubility and stability in the final product. Mixtures of sucrose/glucose syrups continuously form a liquid phase with gelatin [40]. It has been reported that sugars affect the functional properties of proteins, such as adsorption and gelation [49]. However, our study determined that the interactions of sucrose and gelatin with significant models identified for TPA parameters were inverse (Supplementary Table S3). Gummy candies’ TPA analysis is related to parameters such as chewability values, compression force, and energy required for mastication. In tight gels or gel structures, these parameters typically increase. However, the study determined considerably higher chewiness values compared to previous studies. The lower pH value in other studies could be the reason for this. Citric acid is used in some gummy formulations [33]. Citric acid was not used in our investigation for two reasons. Firstly, the possibility that changes in textural properties could be due to the presence of citric acid was considered, along with the effect of promoting the inversion of sucrose, which is one of the independent variables in our study.

In a previous study, Altan Kamer et al. [20] stated that adding sorbitol and sucrose positively affects gelation, texture, thermal, and rheological properties. They mentioned that adding sugars or polyols increases binding regions between proteins and develops a three-dimensional structure. Also, this study found that generally higher sucrose concentrations led to higher chewiness (Supplementary Figure S2), and there was a negligible correlation between the amount of coloring and this parameter. These findings emphasize the importance of sucrose concentration among the factors determining the textural properties of gummy candies.

### 3.3. Color

Color is one of the food’s most essential quality indicators, including confectionery. Its impact on consumers is visual and associated with product variety, quality, and freshness. One of the most significant parameters affecting the sensory perception of confectioneries is their color and visual characteristics. Parameters providing information about the visual attributes of objects include color, brightness, and transparency, which collectively determine the overall perception.

The main color characteristics of the confectionery samples (L*, a*, b*, chroma, and hue angle) and their relationships with the amounts of sucrose, RBEP, and gelatin solution used in the composition were investigated within the scope of the study, and the findings regarding these characteristics are presented in Table 1. The values of L*, a*, b*, chroma, and hue angle were determined in the ranges of 23.9–91.5, (−0.93)–43.6, 1.06–8.17, 6.20–44.0, and 5.97–97.4, respectively. Significant models were detected for all color characteristics except for the b* values, and the R^2^ values of these quadratic models varied between 0.7635 and 0.9991 (Supplementary Table S2). It was determined that the interaction effect of sucrose × gelatin solution on these values was insignificant. However, sucrose × RBEP and gelatin solution × RBEP interactions were significant (*p* < 0.05). Therefore, it can be emphasized that the concentrations of gelatin and sucrose should be considered for the color characteristics of the products obtained using RBEP in gummy confectioneries. The characteristic colors of the products obtained using RBEP were in the red region. The study observed that the increase in sucrose × RBEP had a higher effect on the rise in +a* values, representing this region (Supplementary Figure S2). However, the interactions of both sucrose and gelatin with these color parameters were negligible (Table 2). The correlation of pH values with color parameters, which can generally be described as strong, was noteworthy, showing an increase in L* and hue angle values with increasing pH and a decrease in a* values. In evaluating these results, it should be considered that the optimum pH value for betanin stability is 4.0–5.0 [50].

### 3.4. Antioxidant Activity and Total Phenolics Content

Red beetroot is a commonly used natural colorant due to its betalain content. Additionally, these compounds can be associated with antioxidant activity [51]. Considering the phenolic acid content of red beetroot extract, it gains the characteristic of being a natural colorant source with antioxidant activity [52]. Antioxidant activity (AA) was evaluated by determining the % inhibition values using the DPPH method. The TPC and AA results of the samples were 11.4–257.4 mg GAE/kg and 0.00–49.8%, respectively (Table 3). Significant models were determined for both variables. The R^2^ values for these linear models of TPC and AA were determined as 0.6364 and 0.7289, respectively (Supplementary Table S1). Also, the lack of fit of these models was not significant (*p* > 0.05).

For gummy samples that do not contain RBEP (e.g., Sample 1, 11, and 16), a low amount of TPC was also determined. Although the main source of polyphenols in the gummy composition is RBEP, glucose syrups and sucrose also contain polyphenols at different levels. The TPC values in glucose syrups vary depending on the plant origin and the process used [53]. Polyphenols have also been identified in sucrose derived from sugarcane and sugar beets, as well as in the intermediate products obtained while extracting these products [54]. Additionally, Loncaric et al. [55] noted that the presence of sugar plays a role in enhancing polyphenol extraction.

Protein-polyphenol interactions can be reversible or irreversible [56]. Interactions can affect polyphenols’ physiological properties and activities [57]. Studies have reported positive and negative conditions in polyphenols’ antioxidant properties and bioavailability due to protein-polyphenol interactions. However, no significant correlation was found in this study between the amount of gelatin solution used and AA and TPC (Table 4). This was also applicable to the amount of sucrose (Supplementary Figure S1). However, there was a positive correlation between antioxidant activity and TPC with RBEP concentration (Table 2).

In our study, as expected, AA showed a strong relationship with TPC and increased according to this variable. However, strong relationships were also identified between AA and color characteristics other than chroma. Protein-phenol interactions depend on the structure of phenols and proteins and certain environmental factors (pH, temperature, etc.) [58]. In addition to these parameters, the protein/phenol ratio in the environment is also influential. Interaction with binding sites on the protein chain reaches saturation with low protein concentration, and as the ratio increases, proteins in the colloidal suspension aggregate [23]. This study determined a strong correlation with pH as an environmental factor. A decrease in pH may result in higher antioxidant activity and TPC, possibly due to pH-dependent ionization that influences antioxidant reactions. It was also found that this variable was correlated with RBEP, an independent variable in the study, in the same direction and with similar strength (Table 2).

The ratio of protein/phenolic compounds is an essential factor to be considered in subsequent protein-phenolic interactions. At low protein concentrations, binding sites on the protein chains interact with phenolic compounds until saturation is reached, and as this ratio increases, proteins aggregate into semi-stable colloidal forms [59]. These insoluble protein-phenolic complexes come together, forming large clusters by bridging between saturated polypeptide chains [60]. Conversely, at high protein concentrations, clustering is less because bridges form directly between proteins and phenols before binding sites reach saturation [59]. Thus, more soluble complexes can be formed [60]. These alterations may be among the factors limiting the bioaccessibility of polyphenols. Therefore, future studies investigating the bioaccessibility of polyphenols depending on RBEP and gelatin concentrations would be beneficial.

### 3.5. Storage Stability

Gummy candies prepared with gelatin solution, sucrose, and RBEP at different levels were stored without packaging at 25 °C and 70% relative humidity, a commonly used ambient shelf-life condition. Texture profile analysis (TPA) and color measurements were conducted at 7-day intervals. The study aimed to employ a method compatible with standard practices. The storage stability examination of gummy candies prepared with different gelling agents typically involves simulating normal storage conditions, including humidity levels, equilibrium relative humidity (ERH), and monitoring sensory properties [27]. Table 4 presents the variance ratios in TPA values calculated considering the ΔE values based on color measurements of the samples, with values at the beginning and end of storage under ambient shelf-life conditions considered.

Changes in the texture of gummy candies can occur due to surface crust formation, due to moisture loss, or moisture absorption caused by high humidity in the environment. Eeles et al. [61] monitored the sensory, microscopic, and instrumental properties of jelly candies prepared using different gelling agents (starch, gelatin, starch/gelatin, gum Arabic) under conditions of 20–50% relative humidity and 28–70% relative humidity for 10 weeks. Sensory properties such as chewiness exhibited different effects depending on the moisture content and formulation (gelling agent) used. Due to the ERH values, it was observed that all samples exhibited drying behavior as expected under 20 °C and 50% relative humidity conditions, depending on the moisture content changes. When the changes in hardness value were investigated in this study, an overall increase was observed in all samples. The increase in hardness in candies could be attributed to crystallization, glass transition, or the formation of a gel network by gelatin [37]. Therefore, high-fructose corn syrups are important in controlling sucrose crystallization in confectionery products [62]. However, as previously mentioned, no hardness increase was observed due to sucrose crystallization in the samples. When all samples were examined, the hardness change rates varied widely, ranging from −2.11% to 52.1%. A decrease in the amount of gelatin solution and an increase in sucrose content reduced hardness values with storage changes. A significant quadratic model was determined for this property, with an R2 value of 0.7449 for the model.

Red beet-originated coloring agents are not common in gummy candies. This is due to insufficient storage and processing stability despite being a low-cost coloring agent. Betalains and betacyanins obtained from this plant are more heat-sensitive than other pigments. Colors close to pink can rapidly turn orange-brown tones during processing and storage [22]. Therefore, monitoring the stability of color properties in studies using red beet-derived coloring agents was important. The results of the color changes of samples under ambient shelf-life conditions in this study are presented in Table 4. The ΔE values range from 1.04 to 7.09. The brown color change in the gummies is mainly explained by the oxidation of beet betalains during storage, which becomes faster in the presence of light and oxygen [63]. However, it is noteworthy that numerically, more than 50% of the samples have ΔE values of 3.0 or below. Previous research has suggested different thresholds for ΔE values to determine when color differences are visible to the naked eye. For instance, Faghihi et al. [64], Sakiroff et al. [65], and Lombardelli et al. [66] suggested 3.0, 3.3, 3.7, and 5.0 as critical ΔE values, respectively. No conclusive model could be established to show the impact of independent variables on the ΔE value (Supplementary Table S4). Additionally, no moderate or strong interaction between this parameter and other dependent and independent variables could be identified (Table 2).

### 3.6. Sensory Evaluations

**Figure 1:**
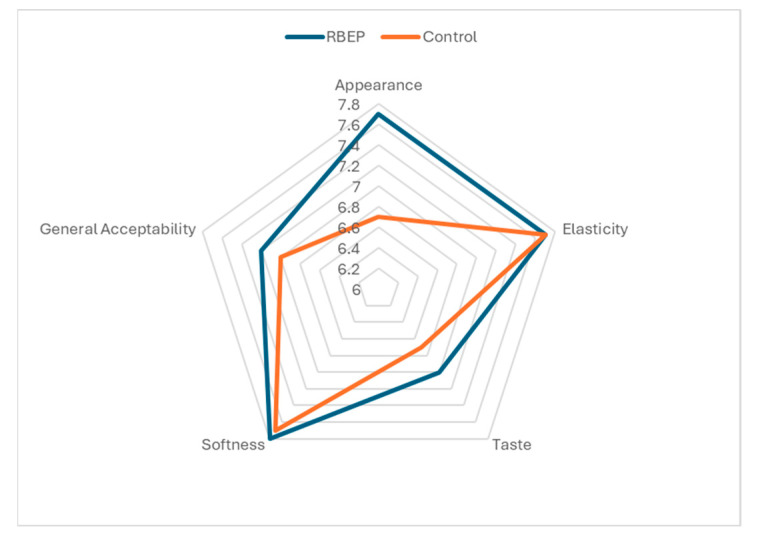
Sensorial evaluation of RBEP-added and not-added gummies at the optimum point indicates insignificant differences (*p* > 0.05) according to the Independent *t*-test.

The composition of the gummy samples was established using ambient shelf-life testing and characterization results. A control sample without colorants was also prepared using the composition commonly used in previous studies [23]. This control sample was produced using the method described in Section 2.3. Sensory analyses were conducted to compare these two samples. Figure 1 displays the sensory characteristics scores of the optimum gummy and control (RBEP-free) samples. Compared to the control samples, the appearance and taste characteristics of the gummy formulation were improved by adding RBEP; nevertheless, the differences were insignificant (*p* > 0.05). Similarly, significant (*p* < 0.05) improvement in the color profile of RBEP-supplemented hard candies was reported by Farhan et al. [67], which was attributed to the presence of natural coloring pigments in beetroot, such as betaxanthins, betacyanins, and betalains. The softness and elasticity were determined to be comparable to the control sample. Although RBEP-added samples’ general acceptability score was 0.2 points higher than the control sample’s, the difference was insignificant (*p* > 0.05). The sensory evaluations demonstrated that incorporating RBEP into gummy formulations showed good acceptance for the gummy confectionery samples.

### 3.7. Optimization Validation

Optimization of gummy samples was carried out by using (i) maximum springiness, (ii) maximum hardness (texture profile analysis), (iii) minimum ΔE* value (ambient shelf-life studies), and (iv) minimum hardness change rate (ambient shelf-life studies). Considering these responses, the optimum composition was 34.52 g/100 g for sucrose, 18.33 g/100 g for gelatin solution, and 0.440968 g/100 g for colorant (Table 5). For the optimum gummy sample, the predicted ΔE value was 3.83. In this study, color stability and change were important parameters due to the investigation of the effects of colorants. Although this value is above the critical limit when considering some previous studies [24] also report a higher critical threshold [65,66]. Additionally, considering the experimental results, the observed ΔE value (2.10) for the optimum gummy sample was lower than the predicted value (3.83) (Table 5).

## 4. Conclusions

The study conducted optimization and modeling using gelatin solution, sucrose, and RBEP as independent variables for gummy candies. The use of RBEP has been determined to significantly affect the final product pH, antioxidant activity, and total phenolic content. Moderate to strong connections between the samples’ hardness, springiness, cohesiveness, chewiness, and water activity properties and their DMC were found. This correlation’s coefficient values ranged from −0.471 to −0.712. An increase in RBEP amount resulted in a significant decrease in pH and a strong and negative correlation (−0.889) existed between pH value and RBEP concentration. It was determined that an increase in sucrose content led to a decrease in hardness and cohesiveness values, while an increase in gelatin content resulted in these parameters. RBEP × gelatin and RBEP × sucrose interactions were significant for hardness change during ambient shelf life. Sucrose × RBEP and gelatin solution × RBEP interactions were significant for all color characteristics except for the b* values. The increase in sucrose x RBEP had a higher effect on the rise in +a* values. The TPC and AA results of the samples were 11.4–257.4 mg GAE/kg and 0.00–49.8%, respectively. Additionally, the significant increase in antioxidant activity in confectionery products is associated with RBEP. For optimization of gummy confectionery production, texture profile analysis (maximum hardness and springiness), ΔE* value determined from ambient storage condition (minimum), and hardness variance ratio, determined from ambient storage condition (minimum), were considered. Based on these responses, the optimal composition for samples prepared with RBEP was determined to be 34.52 g/100 g for sucrose, 18.33 g/100 g for gelatin solution, and 0.44 g/100 g with a 0.725 desirability value. In conclusion, this study identified relationships between RBEP and major components such as sucrose and gelatin concerning various parameters and product characteristic properties. Therefore, RBEP should be used not only based on its visual characteristics and color stability (during processing and storage) but also considering other potential effects.

## Figures and Tables

**Table 1:** Physico-chemical and color properties of RBEP-added gummy candies.

Sample	Sucrose (g/100 g)	Gelatin Solution (g/100 g)	RBEP (g/100 g)	TSS (°Bx)	Dry Matter Content (g/100 g)	Water Activity	pH	L*	a*	b*	C*	h*
1	36.62	16.68	0.00	85.90 ± 0.01	82.57 ± 0.44	0.70 ± 0.03	5.41 ± 0.02	77.10 ± 1.69	−0.05 ± 1.02	8.17 ± 0.64	8.24 ± 0.64	97.20 ± 0.36
2	37.43	15.00	0.87	88.00 ± 0.01	82.77 ± 0.19	0.70 ± 0.01	4.71 ± 0.01	26.70 ± 1.27	24.70 ± 1.72	4.93 ± 0.42	25.20 ± 1.76	11.30 ± 0.57
3	33.55	18.75	1.00	87.70 ± 0.01	80.04 ± 0.29	0.71 ± 0.01	4.87 ± 0.03	23.90 ± 0.43	12.00 ± 0.56	1.79 ± 0.28	12.10 ± 0.59	8.44 ± 0.94
4	36.41	15.89	1.00	89.40 ± 0.01	81.74 ± 0.56	0.67 ± 0.02	4.76 ± 0.02	25.30 ± 1.86	27.10 ± 3.44	5.45 ± 0.59	27.70 ± 3.50	11.40 ± 0.34
5	35.50	17.37	0.43	88.60 ± 0.01	81.37 ± 0.44	0.65 ± 0.01	5.09 ± 0.03	38.70 ± 0.95	41.20 ± 0.65	6.85 ± 0.16	41.80 ± 0.66	9.44 ± 0.14
6	35.50	17.37	0.43	85.00 ± 0.01	80.30 ± 0.35	0.68 ± 0.00	5.19 ± 0.03	26.30 ± 0.88	19.90 ± 0.65	3.98 ± 0.20	20.00 ± 0.91	11.30 ± 0.23
7	35.50	17.37	0.43	84.10 ± 0.01	81.03 ± 0.14	0.71 ± 0.01	5.23 ± 0.03	23.90 ± 0.49	29.40 ± 0.58	6.78 ± 0.39	30.20 ± 0.58	13.00 ± 0.72
8	32.56	19.74	1.00	85.40 ± 0.01	79.85 ± 0.39	0.75 ± 0.02	5.06 ± 0.02	24.90 ± 1.69	10.10 ± 0.41	1.06 ± 0.42	10.20 ± 0.42	5.97 ± 2.29
9	32.00	20.78	0.52	89.20 ± 0.01	80.25 ± 0.13	0.71 ± 0.04	5.23 ± 0.03	31.60 ± 1.19	31.80 ± 1.36	6.19 ± 0.25	32.30 ± 1.39	11.00 ± 0.17
10	37.43	15.00	0.87	87.40 ± 0.01	82.76 ± 0.21	0.66 ± 0.03	4.92 ± 0.03	30.40 ± 1.97	33.20 ± 1.73	6.12 ± 0.63	33.80 ± 1.67	10.50 ± 1.37
11	34.37	18.93	0.00	86.70 ± 0.01	81.40 ± 0.31	0.74 ± 0.01	5.64 ± 0.03	78.60 ± 1.02	−0.52 ± 0.09	6.16 ± 0.69	6.18 ± 0.69	94.80 ± 0.58
12	32.00	20.78	0.52	86.50 ± 0.01	79.98 ± 0.18	0.74 ± 0.03	5.20 ± 0.02	26.60 ± 2.89	11.50 ± 1.47	1.18 ± 1.25	11.60 ± 1.42	8.60 ± 2.67
13	35.50	17.37	0.43	86.10 ± 0.01	81.57 ± 0.46	0.72 ± 0.02	5.40 ± 0.01	43.70 ± 0.45	43.60 ± 0.21	5.91 ± 0.21	44.00 ± 0.24	7.73 ± 0.25
14	33.32	19.98	0.00	85.40 ± 0.01	78.80 ± 0.12	0.72 ± 0.05	5.74 ± 0.02	72.80 ± 1.58	−0.78 ± 0.19	8.09 ± 0.81	7.14 ± 0.94	96.20 ± 0.98
15	34.48	17.82	1.00	84.60 ± 0.01	81.17 ± 0.23	0.74 ± 0.01	5.13 ± 0.02	30.10 ± 0.34	37.50 ± 1.92	8.00 ± 0.49	38.40 ± 1.98	12.00 ± 0.16
16	37.76	15.54	0.00	87.40 ± 0.01	82.79 ± 0.06	0.72 ± 0.03	5.74 ± 0.01	91.50 ± 0.21	−0.93 ± 0.04	6.13 ± 0.12	6.20 ± 0.13	97.40 ± 2.00

Mean ± Standard deviation; C*; Chroma, RBEP; Red Beet Extract Powder; TSS; Total Soluble Solids; L*; luminance, a*; ± red-green, b*; ±yellow-blue, C*; chroma, h*; hue angle.

**Table 2:** Correlation coefficients (r) between variables.

	A: Sucrose	B: Gelatin Solution	C: Coloring Agent	Hardness	Resilience	Cohesion	Springiness	Gumminess	Chewiness	TSS	DMC	aw	pH	L*	a*	b*	C*	Hue Angle	Hardness Variance	∆E	TPC	AA
A: Sucrose	1.000	−0.979	−0.089	−0.617	−0.614	−0.467	−0.009	−0.696	−0.451	0.127	0.871	−0.570	−0.112	0.243	0.122	0.422	0.186	0.185	−0.423	0.023	−0.076	−0.122
B: Gelatin Solution	−0.979	1.000	−0.115	0.669	0.647	0.450	0.067	0.726	0.508	−0.167	−0.863	0.601	0.293	−0.070	−0.227	−0.326	−0.268	−0.019	0.443	−0.074	−0.087	−0.052
C: Coloring Agent	−0.089	−0.115	1.000	−0.265	−0.171	0.073	−0.284	−0.156	−0.288	0.194	−0.028	−0.159	−0.889	−0.842	0.515	−0.463	0.408	−0.815	−0.102	0.253	0.798	0.853
Hardness	−0.617	0.669	−0.265	1.000	0.727	0.364	0.099	0.793	0.665	−0.643	−0.670	0.562	0.343	−0.014	−0.382	−0.196	−0.413	0.139	0.275	−0.107	−0.234	−0.259
Resilience	−0.614	0.647	−0.171	0.727	1.000	0.790	0.533	0.673	0.867	−0.454	−0.712	0.486	0.318	−0.056	−0.283	−0.342	−0.336	0.009	0.248	−0.167	−0.135	−0.162
Cohesion	−0.467	0.450	0.073	0.364	0.790	1.000	0.328	0.528	0.547	−0.164	−0.471	0.343	0.089	−0.169	−0.081	−0.303	−0.138	−0.138	0.119	−0.107	0.118	0.119
Springiness	−0.009	0.067	−0.284	0.099	0.533	0.328	1.000	−0.028	0.755	−0.360	−0.230	−0.030	0.408	0.190	0.004	0.151	0.030	0.120	0.111	−0.290	−0.407	−0.408
Gumminess	−0.696	0.726	−0.156	0.793	0.693	0.528	−0.028	1.000	0.604	−0.442	−0.640	0.582	0.222	−0.137	−0.333	−0.449	−0.398	−0.027	0.145	0.099	0.096	0.097
Chewiness	−0.451	0.508	−0.288	0.665	0.867	0.547	0.755	0.604	1.000	−0.633	−0.630	0.357	0.433	0.023	−0.216	−0.144	−0.239	0.071	0.207	−0.188	−0.252	−0.270
TSS	0.127	−0.167	0.194	−0.643	−0.454	−0.164	−0.360	−0.442	−0.633	1.000	0.283	−0.473	−0.366	−0.060	0.168	−0.080	0.160	−0.135	−0.060	0.326	0.236	0.235
DMC	0.871	−0.863	−0.028	−0.670	−0.712	−0.471	−0.230	−0.640	−0.630	0.283	1.000	−0.402	−0.154	0.233	0.171	0.356	0.230	0.135	−0.478	0.005	−0.032	−0.030
aw	−0.570	0.601	−0.159	0.562	0.486	0.343	−0.030	0.582	0.357	−0.473	−0.402	1.000	0.458	0.233	−0.451	−0.240	−0.467	0.267	0.316	−0.391	−0.129	−0.099
pH	−0.012	0.293	−0.889	0.343	0.318	0.089	0.408	0.222	0.433	−0.366	−0.154	0.458	1.000	0.838	−0.520	0.397	−0.421	0.791	0.266	−0.348	−0.835	−0.837
L*	0.243	−0.070	−0.842	−0.014	−0.056	−0.169	0.190	−0.137	0.023	−0.060	0.233	0.233	0.838	1.000	−0.640	0.492	−0.520	0.960	0.318	−0.331	−0.795	−0.815
a*	0.122	−0.227	0.515	−0.382	−0.283	−0.081	0.004	−0.333	−0.216	0.168	0.171	−0.451	−0.520	−0.640	1.000	0.154	0.987	−0.768	−0.694	0.167	0.408	0.437
b*	0.422	−0.326	−0.463	−0.196	−0.342	−0.303	0.151	−0.449	−0.144	−0.080	0.356	−0.240	0.397	0.492	0.154	1.000	0.300	0.469	−0.196	−0.203	−0.521	−0.581
C*	0.186	−0.268	0.408	−0.413	−0.336	−0.138	0.030	−0.398	−0.239	0.160	0.230	−0.467	−0.421	−0.520	0.987	0.300	1.000	−0.657	−0.703	0.127	0.298	0.320
Hue Angle	0.183	−0.019	−0.815	0.139	0.009	−0.138	0.120	−0.027	0.071	−0.135	0.135	0.267	0.791	0.960	−0.768	0.469	−0.657	1.000	0.438	−0.296	−0.751	−0.794
Hardness Variance	−0.423	0.443	−0.102	0.275	0.248	0.119	0.111	0.145	0.207	−0.060	−0.478	0.316	0.266	0.318	−0.694	−0.179	−0.703	0.438	1.000	−0.161	−0.305	−0.257
∆E	0.023	−0.074	0.253	−0.107	−0.167	−0.107	−0.290	0.099	−0.188	0.326	0.005	−0.391	−0.348	−0.331	0.167	−0.203	0.127	−0.296	−0.161	1.000	0.228	0.240
TPC	−0.076	−0.087	0.798	−0.234	−0.135	0.118	−0.407	0.096	−0.252	0.236	−0.032	−0.129	−0.835	−0.795	0.408	−0.521	0.298	−0.751	−0.305	0.228	1.000	0.973
AA	−0.122	−0.052	0.853	−0.259	−0.162	0.119	−0.408	0.097	−0.270	0.235	−0.030	−0.099	−0.837	−0.815	0.437	−0.581	0.320	−0.794	−0.257	0.240	0.973	1.000

TSS; Total Soluble Solids, DMC; Dry Matter Content, aw; Water Activity, TPC; Total Phenolics Content; AA; Antioxidant Activity, C*; Chroma. r < 0.30; no correlation or weak correlation, 0.30 < r< 0.50; weak correlation 0.50 < r < 0.70; mild correlation, r > 0.70; strong correlation.

**Table 3:** Texture properties, total phenolic content and antioxidant activity of RBEP-added gummy candies.

Sample	Sucrose (g/100 g)	Gelatin Solution (g/100 g)	RBEP (g/100 g)	Hardness (N)	Resilience	Cohesion	Springiness (mm)	Gumminess (N)	Chewiness (Nxmm)	Total Phenolics Content (mg GAE/kg)	Antioxidant Activity (AA, % Inhibition)
1	36.62	16.68	0.00	14.93 ± 0.21	0.59 ± 0.01	0.87 ± 0.01	0.23 ± 0.04	13.02 ± 0.01	2.96 ± 0.01	25.60 ± 0.12	0.00 ± 0.02
2	37.43	15.00	0.87	12.29 ± 1.9	0.69 ± 0.01	0.99 ± 0.01	0.19 ± 0.03	12.15 ± 0.01	2.36 ± 0.01	257.40 ± 0.97	48.50 ± 1.23
3	33.55	18.75	1.00	15.43 ± 1.75	0.79 ± 0.02	1.00 ± 0.01	0.20 ± 0.01	15.49 ± 0.01	3.11 ± 0.01	144.20 ± 0.15	33.50 ± 0.43
4	36.41	15.89	1.00	10.78 ± 1.46	0.57 ± 0.01	0.89 ± 0.01	0.22 ± 0.04	9.62 ± 0.01	2.15 ± 0.01	197.20 ± 0.28	42.30 ± 0.38
5	35.50	17.37	0.43	11.34 ± 0.97	0.78 ± 0.01	0.99 ± 0.01	0.54 ± 0.01	11.18 ± 0.01	6.04 ± 0.01	104.00 ± 0.56	19.00 ± 0.14
6	35.50	17.37	0.43	16.65 ± 0.01	0.82 ± 0.01	0.98 ± 0.01	0.48 ± 0.01	16.32 ± 0.01	7.75 ± 0.01	116.60 ± 0.63	24.60 ± 0.15
7	35.50	17.37	0.43	19.18 ± 0.5	0.88 ± 0.01	1.00 ± 0.01	0.41 ± 0.11	19.14 ± 0.01	7.88 ± 0.01	88.40 ± 0.21	11.20 ± 0.38
8	32.56	19.74	1.00	17.20 ± 0.94	0.89 ± 0.01	0.99 ± 0.01	0.49 ± 0.03	17.05 ± 0.01	8.29 ± 0.01	144.60 ± 0.35	31.80 ± 0.58
9	32.00	20.78	0.52	14.99 ± 0.45	0.76 ± 0.04	1.00 ± 0.02	0.28 ± 0.07	14.94 ± 0.01	4.19 ± 0.01	95.20 ± 0.58	18.70 ± 0.62
10	37.43	15.00	0.87	12.05 ± 0.49	0.64 ± 0.06	0.98 ± 0.03	0.33 ± 0.06	11.81 ± 0.02	3.87 ± 0.01	128.20 ± 0.54	30.50 ± 0.25
11	34.37	18.93	0.00	18.16 ± 1.41	0.83 ± 0.03	1.00 ± 0.02	0.32 ± 0.05	18.11 ± 0.03	5.75 ± 0.01	11.40 ± 0.48	0.00 ± 0.01
12	32.00	20.78	0.52	16.79 ± 0.88	0.84 ± 0.01	1.02 ± 0.01	0.25 ± 0.03	17.16 ± 0.01	4.24 ± 0.01	217.00 ± 1.22	49.80 ± 0.37
13	35.50	17.37	0.43	13.63 ± 1.01	0.66 ± 0.02	0.92 ± 0.01	0.31 ± 0.09	12.53 ± 0.01	3.84 ± 0.01	96.70 ± 0.26	20.30 ± 0.15
14	33.32	19.98	0.00	17.96 ± 1.71	0.87 ± 0.01	1.01 ± 0.01	0.45 ± 0.01	18.11 ± 0.01	8.19 ± 0.01	51.20 ± 0.83	0.00 ± 0.03
15	34.48	17.82	1.00	13.61 ± 1.22	0.73 ± 0.03	1.00 ± 0.01	0.37 ± 0.07	13.62 ± 0.01	5.04 ± 0.01	151.70 ± 0.27	36.90 ± 0.14
16	37.76	15.54	0.00	10.63 ± 0.82	0.75 ± 0.01	0.99 ± 0.00	0.48 ± 0.02	10.53 ± 0.01	5.11 ± 0.01	17.90 ± 0.14	0.00 ± 0.04

Mean ± Standard deviation. GAE, Gallic acid equivalent; RBEP; Red Beet Extract Powder.

**Table 4:** Color differences and texture properties variance ratios of samples under ambient shelf-life conditions.

Sample	Hardness * (%)	Resilience * (%)	Cohesion * (%)	Springiness * (%)	Gumminess * (%)	Chewiness * (%)	ΔEa
1	39.0	46.6	13.5	143.0	57.8	283.2	3.07
2	3.21	13.5	−2.28	19.6	1.00	18.6	1.81
3	46.0	5.17	−3.55	−3.36	40.8	35.7	6.00
4	23.7	38.8	8.96	40.6	35.3	87.7	7.09
5	11.3	2.87	−2.08	−54.0	8.93	−50.0	4.13
6	28.5	1.77	−0.12	−44.7	28.4	−29.0	1.50
7	0.11	−6.63	−2.15	−5.13	−2.04	−6.41	6.36
8	49.6	−4.40	−0.29	−6.94	49.2	38.9	2.43
9	31.4	2.74	−1.14	47.2	29.9	89.7	3.54
10	20.7	−2.47	−3.52	48.9	16.6	72.6	6.70
11	30.8	−3.28	−0.47	35.5	30.2	75.7	1.88
12	9.65	−0.52	−2.31	73.4	7.18	85.5	6.08
13	−2.11	27.1	8.00	88.9	5.84	97.7	3.40
14	52.1	0.27	−1.46	−12.7	49.9	32.0	3.31
15	26.2	3.26	−1.65	12.5	24.1	38.2	1.04
16	32.2	6.07	−1.11	−2.50	30.8	27.3	3.07

*: According to differences between Day 0 and Day 63 at 25 °C, 70%.

**Table 5:** Optimization of Process Conditions and Validation.

Factor A	Factor B	Factor C	Response1	Response 2	Response 3		Response 4
Sucrose (g/100 g)	Gelatin Solution (g/100 g)	RBEP (g/100 g)	Springiness (mm)	Hardness (N)	ΔE* value		Hardness Variation (%)
34.52	18.33	0.44	0.47	15.16	3.83		13.51
**Experimental Results**							
		**95% CI Low for Mean**		**Observed**		**95%CI High for Mean**	
Springiness (mm)		0.18		0.43		0.77	
Hardness (N)		10.36		14.85		19.95	
ΔE value		−0.54		2.10		8.22	
Hardness Variation (%)		10.89		16.22		37.49	

CI: Confidential Intervals.

## Data Availability

The data and materials used in this study are available upon request.

## Supplementary Materials

The following supporting information can be downloaded at: https://www.mdpi.com/article/10.3390/foods14173138/s1. Table S1: ANOVA results for linear and quadratic models of gummy candy samples ‘bioactive and physicochemical properties. Table S2: ANOVA results for linear and quadratic models of gummy candy samples’ color properties and pH value. Table S3: ANOVA results for linear and quadratic models of gummy candy samples’ textural properties. Table S4: ANOVA results for linear and quadratic models of gummy candy samples ’hardness variance and color change during storage. Figure S1: Effect of Sucrose- Gelation Interaction on color, physicochemical and textural parameters of gummy samples. Figure S2: Effect of Sucrose- RBEP Interaction on color, physicochemical and textural parameters of gummy samples. Figure S3: Effect of Gelatin- RBEP Interaction on color, physicochemical and textural parameters of gummy samples.
