# Supplementary material for: Red Beet Extract Powder, Gelatin and Sucrose Interactions in Gummy Candies
Source: Foods. 2025 Sep 8;14(17):3138. doi: 10.3390/foods14173138 (PMC12428044; doi:10.3390/foods14173138)
Supplement: Supplementary file 1 [file foods-14-03138-s001.zip › foods-3836217-supplementary.pdf]

Supplementary Table 1. ANOVA results for linear and quadratic models of gummy candy samples ‘bioactive and physicochemical properties

	Total Phenolic Content (mg GAE/kg)		Antioxidant Activity (%, inhibition)		Water Activity		Total Soluble Solids (°Bx)		Dry Matter Content (g/100 g)	
	SS	*P value*	*SS*	*P value*	*SS*	*P value*	*SS*	*P value*	*SS*	*P value*
Model	47397.16	0.0014	3262.47	0.0002	0.0049	0.0498	9.72	0.3150	17.69	0.0024
X_1	*	0.0014	*	0.0002	*	0.0498	*	0.6362	*	0.0003
X_2	*	0.0014	*	0.0002	*	0.0498	*	0.6362	*	0.0003
X_3	*	0.0014	*	0.0002	*	0.0498	*	0.6362	*	0.0003
X_1X_2	-	-	-	-	-	-	7.41	0.1086	0.7716	0.2064
X_1X_3	-	-	-	-	-	-	-	-	0.0005	0.9475
X_2X_3	-	-	-	-	-	-	-	-	0.0134	0.8623
X_1^2	-	-	-	-	-	-	-	-	-	-
X_2^2	-	-	-	-	-	-	-	-	-	-
X_3^2	-	-	-	-	-	-	-		-	-
Lof	10882.82	0.8681	474.64	0.8793	0.0044	0.6883	14.38	0.6928	3.26	0.1035
P.E	16200.58		738.71		0.0039		15.19		0.9639	
Total	74480.55		4475.82		0.0132		39.30		21.91	
R^2		0.6364	R^2	0.7289	R^2	0.3696	R^2	0.2474	R^2	0.8072
Adj- R^2		0.5804	*Adj-* R^2	0.6872	*Adj-* R^2	0.2726	*Adj-* R^2	0.0593	*Adj-* R^2	0.7108
Pred- R^2		0.3913	*Pred-* R^2	0.5544	*Pred-* R^2	0.0744	*Pred-* R^2	-0.2649	*Pred-* R^2	0.3167
Adeq.Prec.		7.3429	*Adeq.Prec.*	9.5085	*Adeq.Prec.*	5.0551	*Adeq.Prec.*	3.2080	*Adeq.Prec.*	8.3983

*; Linear mixture. The test for the linear mixture terms of the Schffe polynomial model compares the linear coefficient estimates to each other rather than comparing the coefficients to zero. There is no linear effect if the linear coefficients are the same, even though the coefficient estimates may be very large. p value<0.05. X1 = Sucrose (g/100 g), X2 = Gelatin solution (g/100 g), X3 = Red Beet Extract Powder (g/100 g). SS; Sum of squares, Lof; Lack of fit, PE; Pure error, R^2; Determination coefficient, Adj- R^2: Adjusted determination coefficient, Pred- R^2; Predicted determination coefficient, Adeq.Prec; Adequate precision.

Supplementary Table 2. ANOVA results for linear and quadratic models of gummy candy samples' color properties and pH value

	L*		a*		b*		C*		Hue Angle		pH	
	SS	*P value*	*SS*	*P value*	*SS*	*P value*	*SS*	*P value*	*SS*	*P value*	*SS*	*P value*
Model	7928.65	<0.0001	2834.35	0.0062	28.86	0.0548	1842.03	0.0289	22396.11	<0.0001	1.26	<0.0001
X_1	*	<0.0001	*	0.0176	*	0.0548	*	0.0808	*	<0.0001	*	<0.0001
X_2	*	<0.0001	*	0.0176	*	0.0548	*	0.0808	*	<0.0001	*	<0.0001
X_3	*	<0.0001	*	0.0176	*	0.0548	*	0.0808	*	<0.0001	*	<0.0001
X_1X_2	73.54	0.1959	234.28	0.1334	-	-	206.79	0.1632	2.29	0.4304	-	-
X_1X_3	1705.33	<0.0001	1509.57	0.0020	-	-	1018.23	0.0075	36.30	0.0151	-	-
X_2X_3	1548.65	<0.0001	1347.49	0.0029	-	-	889.04	0.0109	41.39	0.0158	-	-
X_1^2	-	-	-	-	-	-	-	-	-	-	-	-
X_2^2	-	-	-	-	-	-	-	-	-	-	-	-
X_3^2	-	-	-	-	-	-	-	-	-	-		
Lof	90.31	0.8885	272.26	0.7996	32.63	0.4821	290.26	0.7889	0.3842	0.7623	0.1936	0.2877
P.E	292.55		605.59		18.62		622.52		18.83		0.0708	
Total	8311.51		3712.20		80.11		2754.81		22415.32		1.53	
R^2		0.9539	R^2	0.7635	R^2	0.3602	R^2	0.6687	R^2	0.9991	R^2	0.8267
Adj- R^2		0.9309	*Adj-* R^2	0.6453	*Adj-* R^2	0.2618	*Adj-* R^2	0.5030	*Adj-* R^2	0.9979	*Adj-* R^2	0.9001
Pred- R^2		0.8986	*Pred-* R^2	0.4501	*Pred-* R^2	-0.0108	*Pred-* R^2	0.2372	*Pred-* R^2	0.9923	*Pred-* R^2	0.7236
Adeq.Prec.		16.7649	*Adeq.Prec.*	6.6085	*Adeq.Prec.*	5.7669	*Adeq.Prec.*	5.2758	*Adeq.Prec.*	64.7194	*Adeq.Prec.*	13.6193

*; Linear mixture. The test for the linear mixture terms of the Schffe polynomial model compares the linear coefficient estimates to each other rather than comparing the coefficients to zero. There is no linear effect if the linear coefficients are the same, even though the coefficient estimates may be very large. p value<0.05. X1 = Sucrose (g/100 g), X2 = Gelatin solution (g/100 g), X3 = Red Beet Extract Powder (g/100 g). SS; Sum of squares, Lof; Lack of fit, PE; Pure error, R^2; Determination coefficient, Adj- R^2: Adjusted determination coefficient, Pred- R^2; Predicted determination coefficient, Adeq.Prec; Adequate precision.

Supplementary Table 3. ANOVA results for linear and quadratic models of gummy candy samples' textural properties

	Hardness (N)		Resilence		Cohesion		Springiness (mm)		Gumminess (N)		Chewiness(Nxmm)	
	SS	*P value*	*SS*	*P value*	*SS*	*P value*	*SS*	*P value*	*SS*	*P value*	*SS*	*P value*
Model	56.11	0.0137	0.067	0.0264	0.0227	0.0401	0.1217	0.5743	69.52	0.0072	43.96	0.0880
X_1	*	0.0137	*	0.0264	*	0.0802	*	0.5743	*	0.0072	*	0.0880
X_2	*	0.0137	*	0.0264	*	0.0802	*	0.5743	*	0.0072	*	0.0880
X_3	*	0.0137	*	0.0264	*	0.0802	*	0.5743	*	0.0072	*	0.0880
X_1X_2	-	-	-	-	0.0002	0.2782	-	-	-	-	-	-
X_1X_3	-	-	-	-	0.0001	0.0323	-	-	-	-	-	-
X_2X_3	-	-	-	-	0.00009	0.0253	-	-	-	-	-	-
X_1^2	-	-	-	-	-	-	-	-	-	-	-	-
X_2^2	-	-	-	-	-	-	-	-	-	-	-	-
X_3^2	-	-	-	-	-	-	-	-	-	-	-	-
Lof	23.08	0.8856	0.0582	0.4518	0.0009	0.4096	0.0388	0.1878	24.31	0.9969	9.43	0.4089
P.E	36.89		0.0311		0.0041		0.0393		41.94		11.85	
Total	116.08		0.1562		0.027		0.1998		135.77		65.23	
R^2		0.4834	R^2	0.4284	R^2	0.7092	R^2	0.0818	R^2	0.5323	R^2	0.3119
Adj- R^2		0.4039	*Adj-* R^2	0.3404	*Adj-* R^2	0.5154	*Adj-* R^2	-0.0595	*Adj-* R^2	0.4604	*Adj-* R^2	0.2061
Pred- R^2		0.2784	*Pred-* R^2	0.1498	*Pred-* R^2	0.0290	*Pred-* R^2	-0.4134	*Pred-* R^2	0.2863	*Pred-* R^2	-0.0185
Adeq.Prec.		6.3612	*Adeq.Prec*	5.7153	*Adeq.Prec*	6.3448	*Adeq.Prec*	1.7784	*Adeq. Prec*	7.0242	*Adeq. Prec*	4.5972

*; Linear mixture. The test for the linear mixture terms of the Schffe polynomial model compares the linear coefficient estimates to each other rather than comparing the coefficients to zero. There is no linear effect if the linear coefficients are the same, even though the coefficient estimates may be very large. p value<0.05. X1 = Sucrose (g/100 g), X2 = Gelatin solution (g/100 g), X3 = Red Beet Extract Powder (g/100 g). SS; Sum of squares, Lof; Lack of fit, PE; Pure error, R^2; Determination coefficient, Adj- R^2: Adjusted determination coefficient, Pred- R^2; Predicted determination coefficient, Adeq.Prec; Adequate precision.

Supplementary Table 4. ANOVA results for linear and quadratic models of gummy candy samples 'hardness variance and color change during storage

	**Hardness Variance (%)**		**ΔE**	
	SS	*P value*	*SS*	*P value*
Model	3098.58	0.0089	8.49	0.8832
X_1	*	0.0559	*	0.6902
X_2	*	0.0559	*	0.6902
X_3	*	0.0559	*	0.6902
X_1X_2	65.21	0.4512	1.36	0.6174
X_1X_3	2128.08	0.0012	1.64	0.5840
X_2X_3	1060.98	0.0017	1.19	0.6409
X_1^2	-	-	-	-
X_2^2	-	-	-	-
X_3^2	-	-	-	-
Lof	267.50	08711	24.09	0.5520
P.E	793.49		27.24	
Total	4159.56		59.81	
R^2		0.7449	R^2	0.1419
Adj- R^2		0.6174	*Adj-* R^2	-0.2872
Pred- R^2		0.4061	*Pred-* R^2	-0.9710
Adeq.Prec.		6.2663	*Adeq.Prec.*	1.7933

*; Linear mixture. The test for the linear mixture terms of the Schffe polynomial model compares the linear coefficient estimates to each other rather than comparing the coefficients to zero. There is no linear effect if the linear coefficients are the same, even though the coefficient estimates may be very large. p value<0.05. X1 = Sucrose (g/100 g), X2 = Gelatin solution (g/100 g), X3 = Red Beet Extract Powder (g/100 g). SS; Sum of squares, Lof; Lack of fit, PE; Pure error, R2; Determination coefficient, Adj- R2: Adjusted determination coefficient, Pred- R2; Predicted determination coefficient, Adeq.Prec; Adequate precision.

Supplementary Figure 1. Effect of Sucrose- Gelation Interaction on color, physicochemical and textural parameters of gummy samples**.**

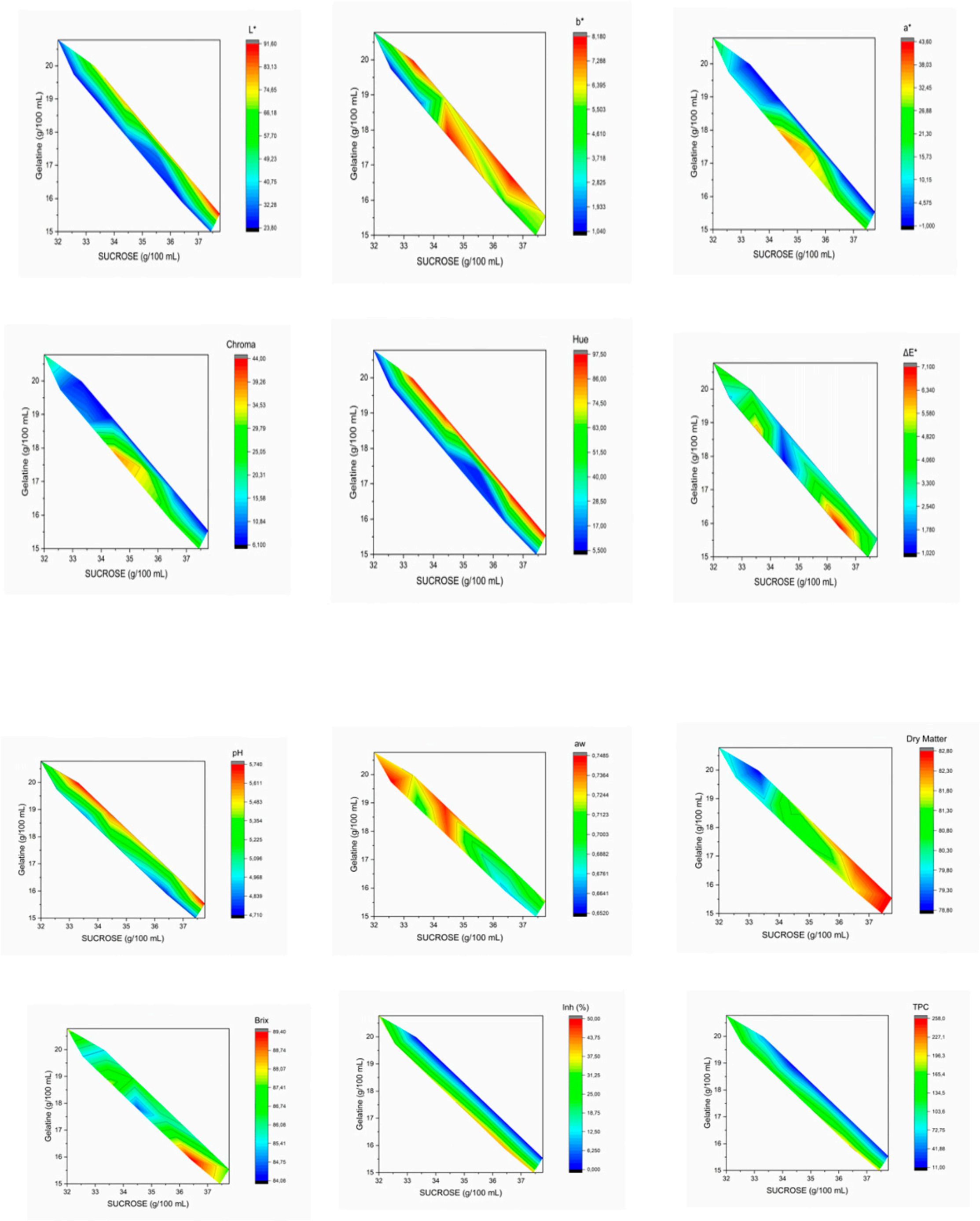

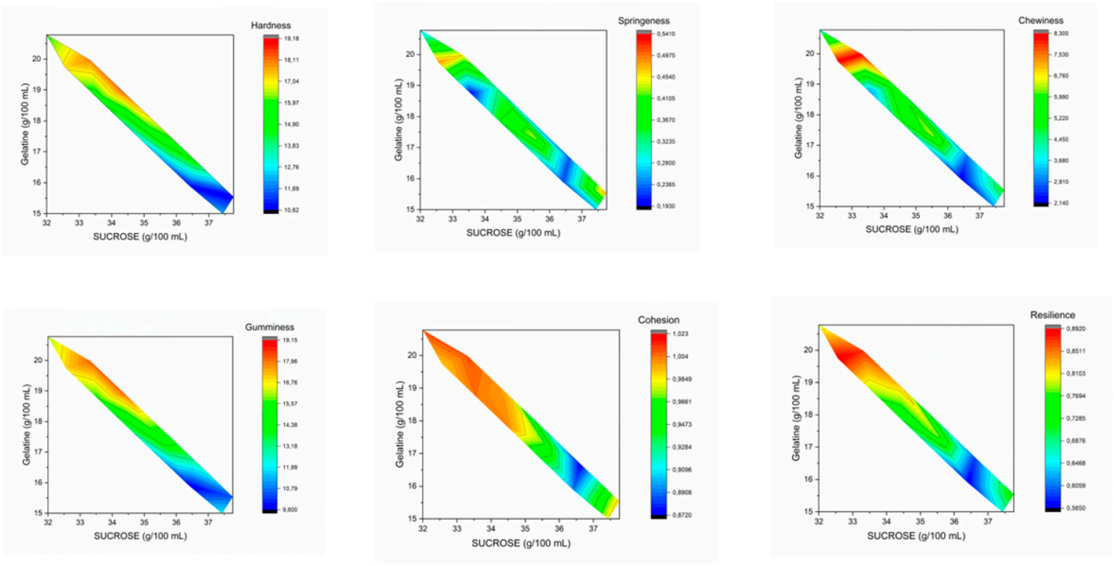

Supplementary Figure 2. Effect of Sucrose- RBEP Interaction on color, physicochemical and textural parameters of gummy samples**.**

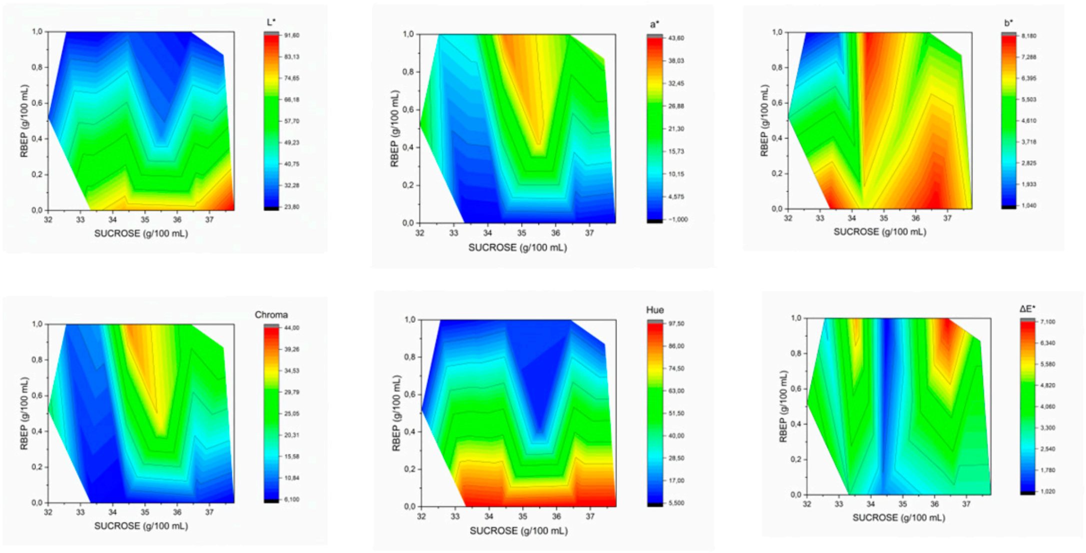

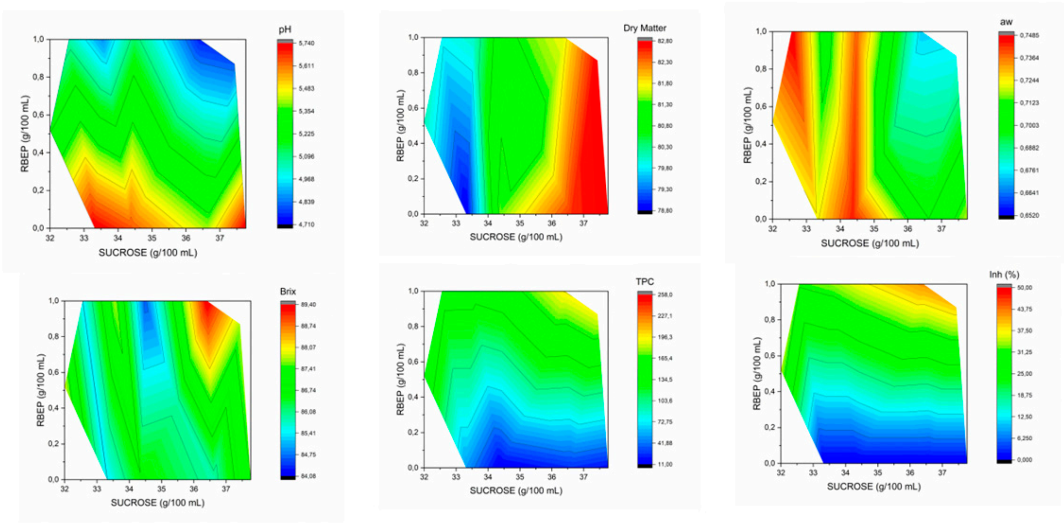
pH
Dry Matter
aw
Brix
TPC
Inh (%)
RBEP (g/100 mL)
SUCROSE (g/100 mL)

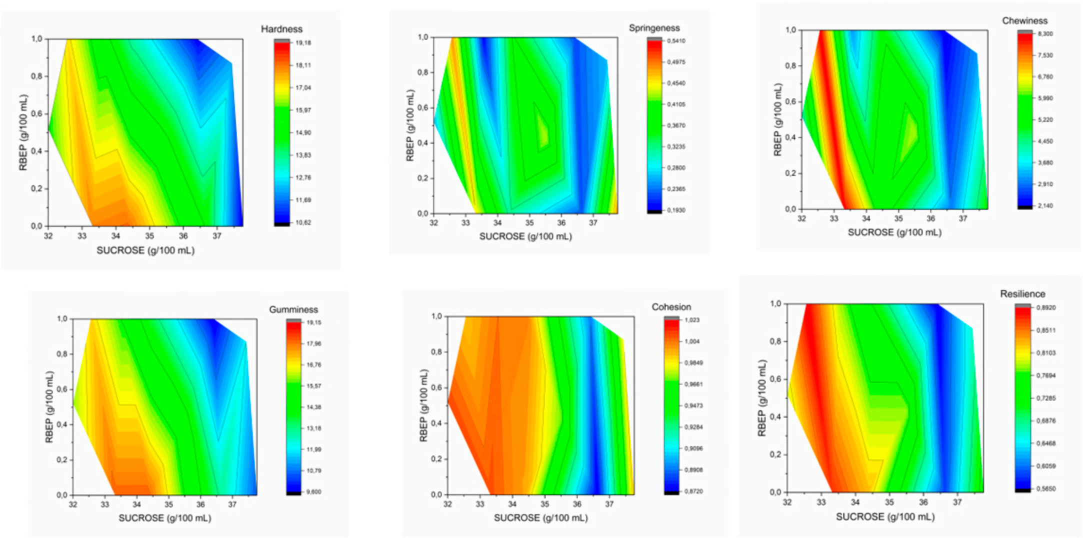
Hardness
Springeness
Chewiness
Gumminess
Cohesion
Resilience
RBEP (g/100 mL)
SUCROSE (g/100 mL)

Supplementary Figure 3. Effect of Gelatin- RBEP Interaction on color, physicochemical and textural parameters of gummy samples**.**

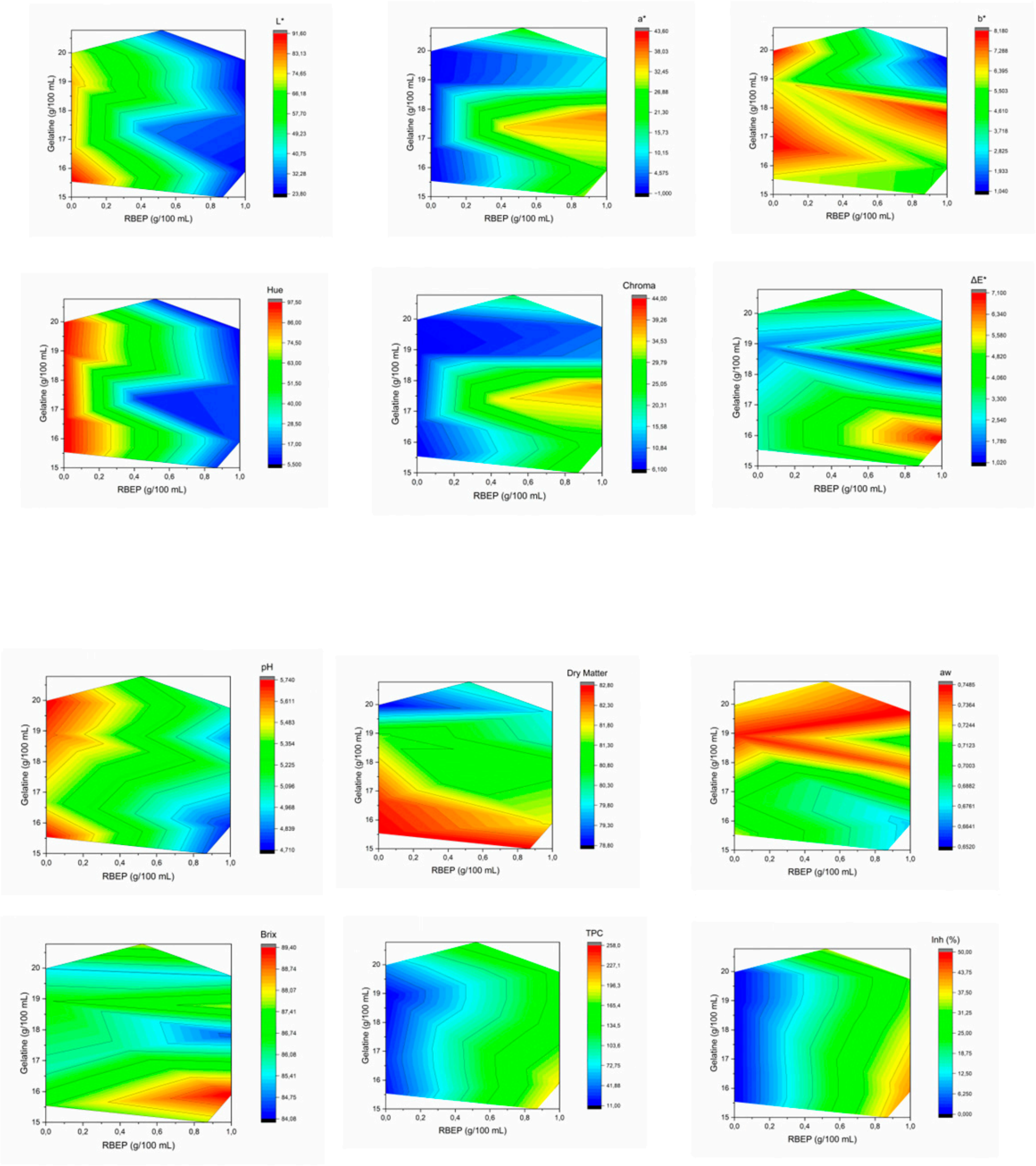

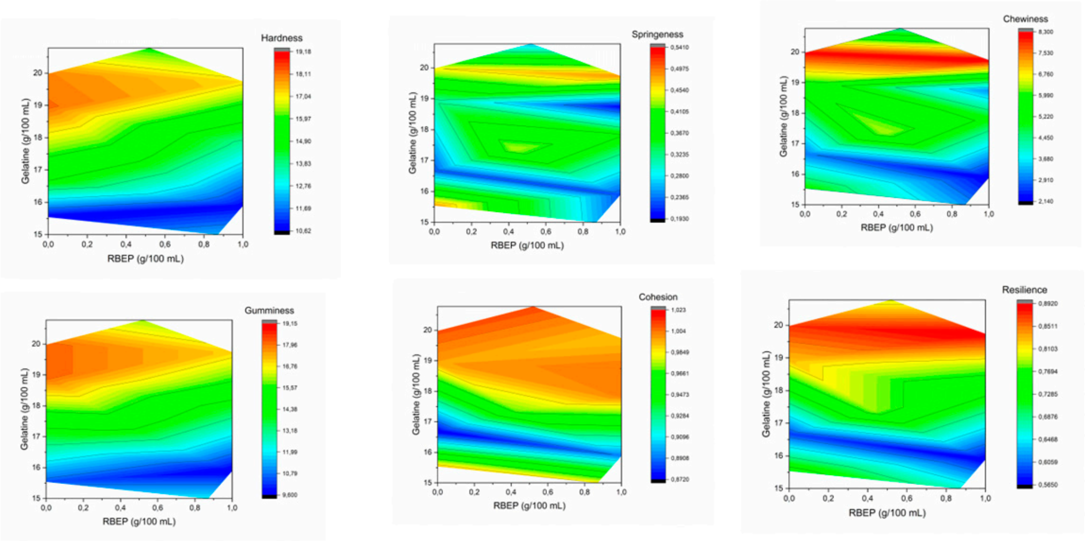

Hardness
19,18
18,11
17,04
15,97
14,90
13,83
12,76
11,69
10,62
Gelatine (g/100 mL)
RBEP (g/100 mL)
Springeness
0,5410
0,4975
0,4540
0,4105
0,3670
0,3235
0,2800
0,2365
0,1930
Gelatine (g/100 mL)
RBEP (g/100 mL)
Chewiness
8,300
7,530
6,760
5,990
5,220
4,450
3,680
2,910
2,140
Gelatine (g/100 mL)
RBEP (g/100 mL)
Gumminess
19,15
17,96
16,76
15,57
14,38
13,18
11,99
10,79
9,600
Gelatine (g/100 mL)
RBEP (g/100 mL)
Cohesion
1,023
1,004
0,9849
0,9661
0,9473
0,9284
0,9096
0,8908
0,8720
Gelatine (g/100 mL)
RBEP (g/100 mL)
Resilience
0,8920
0,8511
0,8103
0,7694
0,7285
0,6876
0,6468
0,6059
0,5650
Gelatine (g/100 mL)
RBEP (g/100 mL)
